# The University of Texas Houston Stroke Registry (UTHSR): implementation of enhanced data quality assurance procedures improves data quality

**DOI:** 10.1186/1471-2377-13-61

**Published:** 2013-06-15

**Authors:** Mohammad H Rahbar, Nicole R Gonzales, Manouchehr Ardjomand-Hessabi, Amirali Tahanan, Melvin R Sline, Hui Peng, Renganayaki Pandurengan, Farhaan S Vahidy, Jessica D Tanksley, Ayodeji A Delano, Rene M Malazarte, Ellie E Choi, Sean I Savitz, James C Grotta

**Affiliations:** 1 Division of Epidemiology, Human Genetics, and Environmental Sciences, The University of Texas School of Public Health at Houston, Houston, TX 77030, USA; 2 Division of Clinical and Translational Sciences, Department of Internal Medicine, The University of Texas-Houston Medical School, Houston, TX 77030, USA; 3 Biostatistics/Epidemiology/Research Design Core, Center for Clinical and Translational Sciences, The University of Texas Health Science Center at Houston, Houston, TX 77030, USA; 4 Department of Neurology, Stroke Program, The University of Texas-Houston Medical School, Houston, TX 77030, USA; 5 The University of Texas Medical Branch at Galveston, Houston, TX 77030, USA

**Keywords:** Stroke, Registry, Quality assurance procedures, Inter-rater reliability, Validity, Quality control, Error rate, Gold standard

## Abstract

**Background:**

Limited information has been published regarding standard quality assurance (QA) procedures for stroke registries. We share our experience regarding the establishment of enhanced QA procedures for the University of Texas Houston Stroke Registry (UTHSR) and evaluate whether these QA procedures have improved data quality in UTHSR.

**Methods:**

All 5093 patient records that were abstracted and entered in UTHSR, between January 1, 2008 and December 31, 2011, were considered in this study. We conducted reliability and validity studies. For reliability and validity of data captured by abstractors, a random subset of 30 records was used for re-abstraction of select key variables by two abstractors. These 30 records were re-abstracted by a team of experts that included a vascular neurologist clinician as the “gold standard”. We assessed inter-rater reliability (IRR) between the two abstractors as well as validity of each abstractor with the “gold standard”. Depending on the scale of variables, IRR was assessed with Kappa or intra-class correlations (ICC) using a 2-way, random effects ANOVA. For assessment of validity of data in UTHSR we re-abstracted another set of 85 patient records for which all discrepant entries were adjudicated by a vascular neurology fellow clinician and added to the set of our “gold standard”. We assessed level of agreement between the registry data and the “gold standard” as well as sensitivity and specificity. We used logistic regression to compare error rates for different years to assess whether a significant improvement in data quality has been achieved during 2008–2011.

**Results:**

The error rate dropped significantly, from 4.8% in 2008 to 2.2% in 2011 (*P* < 0.001). The two abstractors had an excellent IRR (Kappa or ICC ≥ 0.75) on almost all key variables checked. Agreement between data in UTHSR and the “gold standard” was excellent for almost all categorical and continuous variables.

**Conclusions:**

Establishment of a rigorous data quality assurance for our UTHSR has helped to improve the validity of data. We observed an excellent IRR between the two abstractors. We recommend training of chart abstractors and systematic assessment of IRR between abstractors and validity of the abstracted data in stroke registries.

## Backgrounds

Medical registries have been used for many years as sources of clinical data that can support evidence-based medicine and decision-making. Registries are classified according to the disease or disorder, and are defined by patients having the same diagnosis. Stroke is the leading cause of serious, long-term disability and the fourth leading cause of death in the United States [1]. Stroke is the second leading cause of death globally, and all nations, regardless of their health care system, face similar medical and economic burdens [2].

The Harvard Registry is the oldest stroke registry in the US [3,4]. During the last decade, there has been an increased interest in developing other stroke registries to monitor and collect data for improving the quality of care for stroke patients through the assessment of adherence to established performance measures for acute stroke care [5], to study the epidemiology and etiology of specific types of strokes, and to decrease the proportion of premature deaths and disabilities caused by acute stroke. A brief summary of several stroke registries is provided in Table 1.

**Table 1:** Brief summary of several stroke registries in the world

**Stroke registry**	**History and objectives**
Harvard Registry	Oldest stroke registry in the US (1971–1984) [3,4].
Paul Coverdell National Acute Stroke Registry (PCNASR)	Since 2001, with funding from the Centers for Disease Control and Prevention (CDC), the PCNASR was established in collaboration with the state health departments in Georgia, Massachusetts, Michigan, Illinois, North Carolina, Ohio, Iowa, Arkansas, California, New York, and Wisconsin [6-8] with an overall goal of tracking and improving the quality of hospital-based acute stroke care currently available to reduce mortality attributable to stroke, prevent stroke-related disabilities, and prevent recurrent strokes [6].
New England Medical Center Posterior Circulation Registry (NEMC-PCR)	From 1988–1996 the NEMC-PCR thoroughly evaluated all posterior circulation ischemia patients using brain imaging, vascular studies, and appropriate cardiac and hematological investigations to study the epidemiology and etiology of specific types of strokes [9-11].
Get With The Guidelines (GWTG)	Since 2003, the American Heart Association/American Stroke Association has developed a national stroke registry and quality improvement program, known as Get With The Guidelines (GWTG) [5,12-15].
Swedish Stroke Register (Riks-Stroke)	Riks-Stroke was established in 1994 in which patients are followed during the first year after stroke [16].
Registry of the Canadian Stroke Network (RCSN)	RCSN was established in 2001 to allow for the assessment and monitoring of stroke care delivery and outcomes [17].
Australian Stroke Clinical Registry (AuSCR)	AuSCR was established in 2009 to provide national data on the process of care and outcomes for patients who are admitted to hospitals with acute stroke or transient ischemic attack [18].
South London Stroke Register (SLSR)	SLSR is a population based stroke registry that includes stroke patients of all age groups between 1995–1999 [19,20].
Acute Stroke Registry and Analysis of Lausanne (ASTRAL)	ASTRAL is a prospective project designed to assemble state-of-the-art data for all ischemic stroke patients hospitalized in the only stroke unit in the wider area of Lausanne, Switzerland which was initiated in 2002 [21].
Austrian Stroke Unit Registry	Since 2003, this registry is administered by the Gesundheit Osterreich GmbH (Health Austria GmbH) in which 26 out of the 32 existing stroke units in Austria take part [22].
Arbeitsgemeinschaft Deutscher Schlaganfall-Register (ADSR)	Established in 1999, ADSR was developed by the German Stroke Registries Study Group that has defined a "Minimum Dataset" for the evaluation of quality indicators of stroke treatment in Germany in which six regional stroke registries collaborate [23,24].
Danish stroke registry and contribution to the World Health Organization Monitoring Trends and Determinants in Cardiovascular Disease (WHO MONICA) Project	During 1982–1991, within the Glostrup Population Studies in Copenhagen County, a Danish stroke registry was established with the objective of monitoring stroke events in the community over a 10-year period and contributing data to the World Health Organization Monitoring Trends and Determinants in Cardiovascular Disease (WHO MONICA) Project [25,26].
China National Stroke Registry	Since 2007, the China National Stroke Registry recruited consecutive patients with diagnoses of acute cerebrovascular events from 132 hospitals that cover all 27 provinces and four municipalities (including Hong Kong) in China [27].
Taiwan Stroke Registry (TSR)	Since 2006, TSR is sponsored by the Taiwan Department of Health that involves 39 academic and community hospitals and covers the entire country. TSR is the first nationwide effort in Taiwan to establish a reliable national stroke database for assessing the quality of stroke care and identifying areas that require improvement [2].
Japanese Standard Stroke Registry Study (JSSRS)	Since 1998, this registry has accumulated records from 163 Japanese institutions [28].

Despite availability of many stroke registries, limited information has been published regarding standard procedures to ensure reliability and validity of data in stroke registries [5,16,18,29-31]. For example, Reeves *et al*. (2008) reported data regarding reliability of abstracted data collected during 2001–2004 from Michigan PCNASR [29]. Recently, Xian *et al.* (2012) reported data regarding validity of data in the GWTG registry indicating a high level of accuracy for select variables including age, diagnosis, arrival date/time, tPA therapy when compared with audited data from medical records [5]. However, information regarding development and implementation of standard procedures to ensure data quality for stroke registries is limited, particularly at various stages of data management including chart abstraction and quality control of data.

Since 2001, the Specialized Program Of Translational Research in Acute Stroke (SPOTRIAS), funded by the National Institute of Neurological Disorders and Stroke (NINDS), supported the development of prototype registries, which were led by academic principal investigators and medical institutions, to collect data on the quality of care provided to stroke patients from the initial emergency response to hospital discharge. Currently, there are eight funded SPOTRIAS sites that collaborate on this effort. The Stroke Program at the University of Texas Health Science Center at Houston (UTHealth) is part of the SPOTRIAS network [32].

The UTHealth stroke program has played a significant role in the treatment and prevention of stroke and is committed to high quality research, clinical practice, education, and optimal implementation of thrombolysis therapy following acute stroke in Houston, with thrombolytic treatment rates exceeding 30%. As a major stroke center, the Memorial Hermann Hospital-Texas Medical Center, Houston (MHH-TMC) has served as a leader in stroke research for some of the most important acute stroke studies in the world, including the NINDS tissue plasminogen activator (tPA) trial, which led to the approval of the clot-dissolving drug tPA in the treatment for acute stroke [33]. These achievements contributed to our success in being selected as a SPOTRIAS site.

The UTHealth SPOTRIAS data core is responsible for data abstraction, data entry, quality control, statistical analysis, and management of data for the UTHealth Stroke Registry (UTHSR) and other clinical trials. The data core has invested a significant amount of effort to improve the quality of the data in UTHSR. During the past 10 years, we have gained significant insight regarding the design, development, maintenance, quality control, and utilization of our stroke registry. The purpose of this article is to describe the development and assessment of enhanced quality assurance (QA) procedures in UTHSR and compare data quality in UTHSR before and after implementation of our enhanced QA procedures.

## Methods

### History of Houston stroke program and development of UTHSR

**Figure 1:**
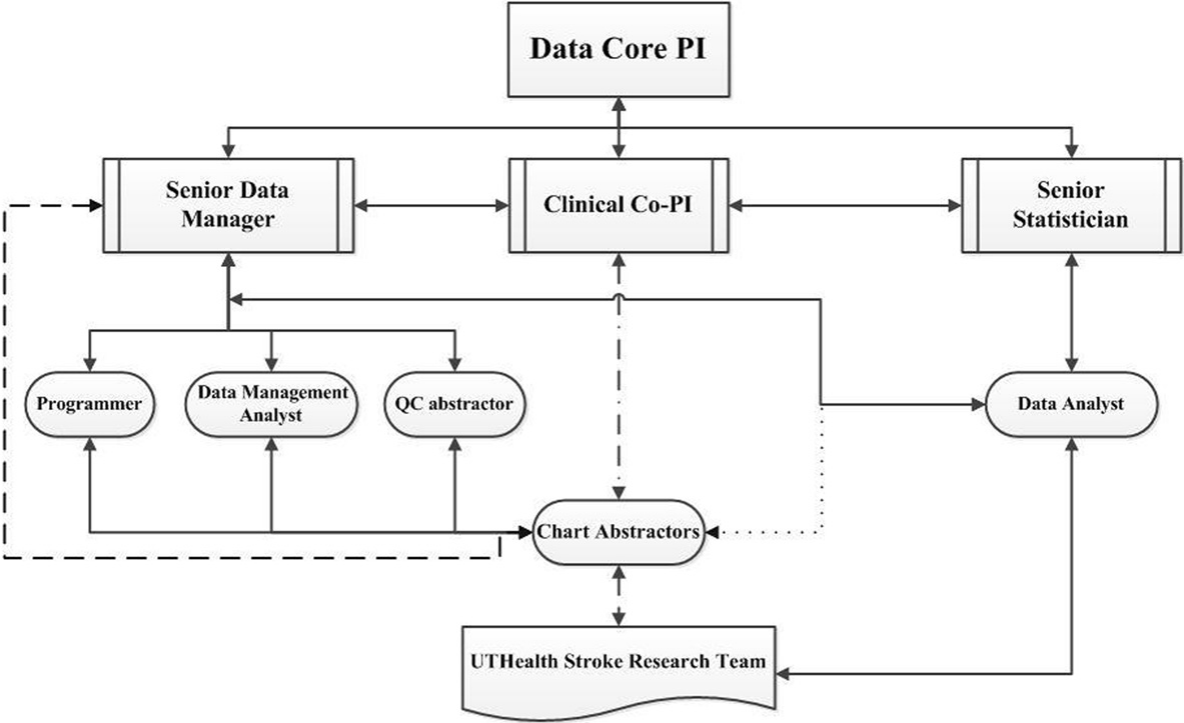
Organizational chart for UTHealth Stoke Data Core.

Houston is the fourth most populated city in the US and is home to the largest medical center in the world [34]. MHH-TMC was the first hospital in the Texas Medical Center. The Neurology department at MHH-TMC was one of the first in the US to use tPA for acute stroke and it was also the first hospital named as a “primary stroke center” by the state of Texas [35]. Historically, the stroke program at UTHealth formed in 1979 with the recruitment of Dr. Grotta. In 1986, the stroke team began to keep a written “log” of all patients admitted to the stroke service. Once the UTHealth stroke team started testing tPA in 1989, they began to keep slightly more detailed records. Once tPA was approved in 1996 [36], the data collected pertaining to tPA use became even more detailed in the stroke database leading to some of the UTHealth stroke team’s earlier publications. In 2002, the SPOTRIAS P50 mechanism funded a data core, which prompted the UTHealth stroke team to convert to an electronic database. In 2003, the UTHealth stroke team began to design and record data in UTHSR for all patients admitted to the UTHealth stroke service at MHH-TMC. In 2008, with the second round of SPOTRIAS funding, the data core made a specific commitment to develop and implement enhanced strategies for improving data quality in UTHSR. The data core represents a collaborative team of investigators supported by data managers, statisticians, programmers (system and web developers), two chart abstractors, and a quality control abstractor. An organizational chart that demonstrates the role and working relationships among various members of the Data Core is provided in Figure 1.

### Design of UTHSR, data elements, and data sources

Attributes considered in designing a registry must ensure that data are valid, reliable, responsive, interpretable, and translatable [37]. UTHSR is a prospective registry initially designed to capture essential information on all patients admitted to the UTHealth in-patient stroke service at MHH-TMC, with the primary aims of tracking the number of patients treated with intravenous (IV) tPA, their essential demographics, and complication rates, and to support research by members of the stroke team. With the funding of SPOTRIAS, the Principal Investigators (PIs) of the original SPOTRIAS sites decided to obtain common data elements that described essential demographics of all patients treated with IV tPA or enrolled in any clinical trials. UTHSR was consequently expanded to incorporate other elements including those variables that were needed for clinical trials that were conducted by the UT stroke team and variables that were needed for reporting to The Joint Commission (TJC) [38,39], as well as select variables to meet minimum requirements for reporting to Centers for Medicare and Medicaid Services (CMS) as they pertain to the vascular neurology aspects of required reporting [40]. All patients who have been admitted to the stroke unit at MHH-TMC are classified by stroke diagnosis subtypes, including infarct (non-hemorrhagic stroke), intracerebral hemorrhage (ICH), intraventricular hemorrhage (IVH), transient ischemic attack (TIA), subarachnoid hemorrhage (SAH), epidural hematomas (EDH), subdural hematomas (SDH), non-acute infarct, and others that could not be classified as any of the above (“Not stroke”), and are entered in UTHSR. Other data elements include admission information (e.g., arrival date and time), medical history, National Institutes of Health Stroke Scale (NIHSS), modified Rankin Scale (mRS) score, Glasgow Coma Scale (GCS), laboratory results, CT scan, CT scan angiogram, MRI, MR angiogram images, thrombolysis therapy (e.g., tPA time and door to needle time), intra-arterial therapy (IAT), complications, and discharge information including: death, mRS on discharge (or day 7, whichever comes first), discharge disposition (home, skilled nursing facility, etc.), and particularly patient education and mRS at 90 days. Currently, the UTHealth stroke team captures up to 235 variables for each patient depending on stroke subtypes. Since some of these variables have multiple responses (e.g., medical history), the number of fields in UTHSR is 372. As UTHSR is modified, corresponding changes to the codebook are made; the codebook is also updated periodically as changes to the abstraction rules are identified or where clarity can be improved. The data core has developed policies for documentation. Members of the data core are responsible for adhering to all policies and procedures established.

### Data sources and data entry into UTHSR

The most important source for data abstraction is the MHH-TMC electronic medical records (EMR) that includes all related personal and medical information. All registry data are manually abstracted from electronic chart review and from rounding with the stroke team. Our abstractors review the entire chart and capture the required information for each patient from admission to discharge from the stroke service. Ambiguous and questionable data, particularly from complicated cases, are discussed in weekly meetings whose regular attendees include vascular neurology UTHealth faculty members involved in the stroke program. In addition, the data core holds weekly meetings to give the abstractors an opportunity to discuss issues related to UTHSR and the abstraction of data. At first, abstraction was carried out by stroke research nurses and fellows, but after SPTORIAS funding, we hired dedicated full-time abstractors who are key members of the data core. Requirements are familiarity with medical terminology and willingness to stay for at least 1 year; our abstractors are usually health care professionals or other medical personnel. Abstractor training will be described later, but is a critical part of the quality assurance component of UTHSR. UTHSR codebook serves as the protocol for data abstraction. Since registries must confidentially maintain patients’ health information, security is an important issue [37]. We maintain confidentiality during all phases of data abstraction, monitoring, analysis, dissemination, and publication. The UTHealth servers reside in a secure location with limited access. All data are automatically backed up daily with redundant storage in a protected off-site location in accordance with UTHealth policies.

### Data quality assurance procedures

QA steps include establishment and implementation of procedures that ensure the quality of data from the point of abstraction to analysis [41]. Strategies and procedures that help to improve data quality in stroke registries include training of abstractors [42], assessment of reliability (e.g., inter-rater reliability (IRR) between abstractors) [5,29,30], and validity of data [43]. After data cleaning [44] and resolution of potential discrepancies, an assessment of validity of data (e.g., accuracy level or error rate) based on a sample of re-abstracted or audited records in the registry is done. For UTHSR, our QA procedures mainly focus on the training of data abstractors, assessment of IRR between abstractors, development and implementation of formal data cleaning procedures, and evaluation of validity of data by calculating error rate or accuracy rate and other measures of validity (e.g., sensitivity, specificity), as will be described here.

#### Data abstractors’ training

For UTHSR, each abstractor is trained by using a codebook (or data dictionary) because a good understanding of the variables in the registry and where to locate their appropriate values by abstractors is essential to ensure data quality. The training of data abstractors also includes re-abstraction of data for a set of patients whose records are already available in UTHSR. Once an abstractor has demonstrated a reasonable level of confidence in abstraction, we provide a new set of patients for abstraction. During this phase, the abstractors attend rounds on the stroke clinical service and enter data in the registry under the supervision of a more experienced abstractor. Abstractors are also trained in contacting the discharged patients to obtain the 90-day mRS data.

#### Reliability study

Reeves *et al.* (2008) have demonstrated assessment of inter-rater reliability to establish the reliability of abstraction between two abstractors (hospital abstractors and the audit abstractor) [29]. In this study, for evaluating IRR between two abstractors, we randomly selected 30 patient records between 2008–2011 and asked two abstractors to re-abstract select variables including initial presentation, arrival time, age, INR, stroke type, onset time, tPA therapy, tPA time, symptomatic hemorrhage, mRS on discharge, and disposition at discharge. For continuous variables, IRR is assessed through the intra-class correlation coefficient (ICC) [29]. For categorical variables, IRR is assessed using the Kappa statistic [29,42]. Since the IRR measures alone are not sufficient to assess the consistency of the abstraction, for binary variables, we assessed the Bias Index (BI) as defined by Reeves *et al.* (2008) [29]. For continuous variables, we calculated mean differences to assess the BI. We also provide 95% confidence intervals for the IRR measures.

#### Data cleaning

Data cleaning refers to a set of processes that involve identification and resolution of all discrepant data, including missing values, incorrect or out-of-range values, or implausible responses that are logically inconsistent with other responses in the database [37,44]. Establishing standard data cleaning processes helps to detect and correct errors, resulting in higher data quality [45]. Since 2003, UTHSR has undergone a series of changes including the development and implementation of additional data quality checks in our data collection system program for prevention of data entry errors. In addition, we have developed and implemented data cleaning, including about 350 univariable and multivariable rules that detect potential data inconsistencies (invalid missing, impossible and implausible). Invalid missing fields are defined as those where the information should have been collected but was not collected or not entered in the registry. Impossible data are defined as data entries that are invalid, do not comply with the codebook, or are out of range. Implausible data are defined as those that are logically inconsistent with data in other fields or seem to be unusual based on statistical rules (e.g., Chebyshev’s rule). Chebyshev’s rule states that for random variables with finite variance, no more than 4% of the data can exceed more than five standard deviations to the right or left of the mean of the distribution. This rule helps to identify potential outliers regardless of the shape of the distribution [46,47]. For some variables, we check the measurements above the 90^th^ and below the 10^th^ percentiles. Based on these rules, the data management analyst prepares a list of invalid missing, impossible, and implausible data for the abstractors so they can double check their entries and resolve potential errors. Once these issues are addressed, the data manager will rerun the same program to confirm that all issues are resolved.

#### Validity study

Validity of captured data by abstractors in a registry against a set of audited medical records or a “gold standard” provides an assessment of quality of data abstracted [43]. Xian *et al.* (2012) reported an overall composite accuracy rate of 96.1% for all data elements in the GWTG-Stroke registry [5]. Riks-Stroke registry also reported at least 95% consistency between the medical chart vs. what is recorded in Riks-Stroke registry for stroke subtype and clinical data, but much lower (approximately 85%) for data related to the health-care organization at the participating hospitals [16]. We have established a rigorous process for assessing validity of data in UTHSR. For this purpose, we randomly selected 115 patient records from UTHSR that consisted of the 30 patient records (with selected variables that were used for the reliability study) and another set of 85 patient records for which our quality control (QC) abstractor re-abstracted data for all variables by reviewing medical record charts and hospital records. These data were adjudicated by a team of experts in the data core that included a vascular neurologist (faculty or fellow), herein called the “gold standard”. These 115 patient records resulted in a total of 8877 data entries or data points in UTHSR during 2008–2011. We calculated the proportion of discrepant data elements relative to the number of entries re-abstracted (i.e., 8877). This error rate could be a combination of data entry in the registry and error in abstraction. For binary variables, we also assessed sensitivity and specificity as shown in Reeves *et al.* (2011) [31,43]. Since sensitivity and specificity are not applicable for continuous variables, we calculated differences between the two sets of data for these variables. Agreement for these variables is defined as when there is no difference between the two set of values. We also calculated mean difference for each variable between the two sets of data. In addition, since we had the “gold standard” for the 30 patient records that were included in the reliability study, we assessed validity of data abstracted by each of the two abstractors involved in the reliability study.

##### Statistical analysis

We conducted descriptive analyses to provide summary statistics for certain key variables in UTHSR. For continuous variables with normal distributions, we used means (Standard Deviation (SD)) but when there was a significant departure from normality (e.g., skewed distributions) we used medians (Interquartile Range (IQR)). We calculated performance measures of care for stroke patients (STK-1 to STK-10) [48,49] in UTHSR based on eligibility of patient related to diagnoses of ischemic stroke and hemorrhagic stroke. We used the Kappa statistic to estimate IRR for nominal variables along with 95% lower confidence limits (LCL) for IRR [50]. For continuous and ordinal variables, we used ICC with a 2-way, random effects ANOVA model [29]. In addition, we calculated the bias index (BI) [29] between data re-abstracted by the two abstractors. For binary variables, the bias index ranges between −1 and +1 with zero indicating no bias [29]. For continuous variables we used mean difference between the two set of values compared as the bias index. When the two abstractors are compared, a positive or negative BI indicates bias between the abstractors [51]. However, when each abstractor is compared with the “gold standard”, a positive or a negative BI shows that the distribution of values produced by the abstractor is shifted to the right or left of the “gold standard”, respectively.

For the validity study, as mentioned earlier we assessed level of agreement, sensitivity, and specificity along with 95% confidence intervals (CIs). Finally, we used logistic regression to compare error rates between 2008 and subsequent years. We also calculated 95% CIs for error rates in different years. All comparisons were made at 5% level of significance. All analyses were conducted using SPSS, Version 20 [52] or SAS software, Version 9.3 [53].

## Results

Descriptive analysis of our total registry data indicated that the mean age of patients was 62.7 years (SD = 15.8); 50.2% were male; 31.7% were African-American; 48.3% were Caucasian; and 14.1% were Hispanic. The distribution of stroke subtypes included 51.4% infarcts and 24.6% hemorrhage stroke (ICH, SAH, EDH, SDH, and IVH).

About 61% of the patients arrived at the hospital by ambulance and nearly 22% were transported by air. Nearly 39% were transferred patients from other hospitals. Approximately 37% of patients who had infarct reported an onset time of less than 2 hours prior to presentation. For patients with infarct, the mean time from arrival to thrombolysis treatment time was 68 minutes (including patients treated with off-label use of thrombolytic). The overall in-hospital mortality among the stroke patients was 8.2% (ischemic stroke 5.8%, ICH 21.4%). The median discharge mRS was 4.0 for all discharged patients. Additional information regarding demographics and other characteristics of the patients at hospital arrival and discharge are reported in Table 2.

**Table 2:** Summary characteristics of patients at arrival and discharge time in UTHSR, 2008–2011 (N=5093)

**Demographics and characteristics of patients (N=5093)**		**n**	**%**	
**Race/Ethnicity**	Caucasian	2459	48.3	
	African-American	1616	31.7	
	Hispanic	719	14.1	
	Asian	161	3.2	
	Other	126	2.5	
	Unknown	12	0.2	
**Medical history**^a^	Hypertension	3464	68.0	
	Type II diabetes	1375	27.0	
	Atrial fibrillation	595	11.7	
	CAD/MI^b^	838	16.5	
	Hyperlipidemia	1316	25.8	
	Prior stroke	1610	31.6	
**Stroke subtype (diagnosis at discharge)**	Infarcts (both infarct and non–acute infarct)	2619	51.4	
	Hemorrhage^c^	1254	24.6	
	Transient ischemic attack (TIA)	319	6.3	
	Not stroke	900	17.7	
	Unknown	1	0.0^d^	
	**All patients admitted to MHH-TMC**^e^			
		**All patients (N=5093)**	**Infarct**^f^**(N=2572)**	**ICH (N=1198)**
**Hospital arrival mode, n (%)**	Ambulance	3113 (61.1)	1580 (61.4)	796 (66.4)
	Air	1146 (22.5)	604 (23.5)	342 (28.6)
	Private vehicle	577 (11.3)	254 (10.0)	15 (1.3)
	Other	120 (2.4)	84 (3.3)	16 (1.3)
	Unknown	137 (2.7)	50 (1.9)	29 (2.4)
**Initial presentation,n (%)**	MHH-TMC in-hospital stroke	110 (2.2)	83 (3.2)	11 (1.0)
	MHH ED	2985 (58.6)	1627 (63.3)	479 (40.0)
	Transfers	1998 (39.2)	862 (33.5)	708 (59.0)
**NIHSS on admission, median (IQR)**		7 (2~16)	7 (3~15)	14 (5~28)
**GCS on admission, median (IQR)**		15 (11~15)	15 (12~15)	12 (6~15)
	**MHH-TMC ED stroke admissions**			
		**All stroke MHH EDpatients (N=2984)**	**Infarct**^f ^**(N=1627)**^g^	**ICH (N=479)**^h^
**Symptom onset to presentation time, n (%)**	≤ 2 hr	1060 (35.5)	597 (36.7)	228 (47.6)
	≤ 3.5hr	1353 (45.3)	753 (46.3)	271 (56.6)
**Arrival to CT time (minutes), median (IQR)**		34 (22~63)	33 (21~57)	29 (19~42)
**Door to needle (tPA) time**^i^**(minutes), median (IQR)**		69 (53~93)	68 (52~90)^k^	NA^j^
	**Stroke patients transferred to MHH-TMC from other hospitals**			
		**All transferred patients (N=1998)**	**Infarct**^f^**(N=862)**^g^	**ICH (N=708)**^h^
**Received tPA and transferred to MHH, n (%)**		276 (13.8)	231 (26.8)	NA^j^
	**Characteristics of stroke patients at discharge from MHH-TMC**			
		**All patients (N=5093)**	**Infarct**^f^**(N=2572)**	**ICH (N=1198)**
**Death, n (%)**		417 (8.2)	150 (5.8)	256 (21.4)
**mRS on discharge, median (IQR)**		4 (2~5)	4 (2~4)	4 (3~5)
**mRS 0–1 on discharge, n(%)**		1052 (20.7)	445 (17.3)	86 (7.2)

^a^ Some patients had more than one prior medical condition; ^b^ CAD (coronary artery disease)/MI (myocardial infarction); ^c^ Hemorrhage category is primarily spontaneous ICH, but also includes primary IVH and rare cases of SAH, EDH, SDH;^ d^ The original value was 0.02%, after rounding it was changed to 0.0%;

^e^*MHH-TMC* = Memorial Hermann Hospital, Texas Medical Center, Houston; ^f^ Infarct does not include non–acute Infarct; ^g^ The sum of infarct patients in the stratified (or subgroup) analyses do not add up to 2572, because 83 patients were admitted to the stroke service from other units of MHH-TMC;^ h^ The sum of ICH patients in the stratified (or subgroup) analyses do not add up to 1198 because 11 patients were admitted to the stroke service from other units of MHH-TMC; ^i^ Door to needle (tPA) time includes off-label tPA treatment;^***j***^*NA* = Not applicable; ^k^ This includes patients treated with off-label thrombolysis.

Overall, during 2008–2011, 32.2% of patients presenting to our emergency department (ED) with acute cerebral infarct received tPA within 4.5 hours of symptom onset and 24.1% received tPA within 3 hours of symptom onset. The data indicate a significant upward trend in the proportion of patients who received tPA between 2008–2011 (*P*-value based on Chi-square test for linear trend < 0.02). Other data related to the tPA times are presented in Table 3.

**Table 3:** Distribution of “Onset to tPA Time” among infarct patients presenting at MHH-TMC, 2008–2011 (N=1627)

**Onset to tPA Time**		**Year**					
		**2008**	**2009**	**2010**	**2011**	**Total**	P-value*
		**n (%)**	**n (%)**	**n (%)**	**n (%)**	**n (%)**	
**Received tPA**		130 (30.4)	124 (34.6)	153 (38.5)	193 (44.5)	**600 (36.9)**	**-**
**No tPA**		297 (69.4)	232 (64.6)	243 (61.2)	247 (55.8)	**1019 (62.6)**	**-**
**Unknown**		1 (0.2)	3 (0.8)	1 (0.3)	3 (0.7)	**8 (0.5)**	**-**
**Total** # **of infarcts**		**428**	**359**	**397**	**443**	**1627**	**-**
**tPA**	0 min - 1 hr	1 (0.2)	0 (0.0)	0 (0.0)	1 (0.2)	**1 (0.1)**	**-**
	61 min - 2 hr	30 (7.0)	34 (9.5)	48 (12.1)	48 (10.8)	**160 (9.8)**	**-**
	121 min - 3 hr	61 (14.3)	40 (11.2)	58 (14.6)	72 (16.2)	**231 (14.2)**	**-**
	181 min - 4.5 hr	19 (4.4)	37 (10.3)	33 (8.3)	42 (9.5)	**131 (8.1)**	**-**
	271 min - 6 hr	5 (1.2)	4 (1.1)	2 (0.5)	12 (2.7)	**23 (1.4)**	**-**
	> 6 hr	15 (3.5)	9 (2.5)	12 (3.0)	18 (4.1)	**54 (3.3)**	**-**
**Received tPA (Total)**		**130**	**124**	**153**	**193**	**600**	**-**
**Within 2 hrs.**		30 (7.24)	34 (9.5)	48 (12.1)	49 (11.1)	**161 (9.9)**	0.02
**Within 3 hrs.**		91 (21.3)	74 (20.6)	106 (26.7)	121 (27.3)	**392 (24.1)**	0.01
**Within 4.5 hrs.**		110 (25.7)	111 (30.9)	139 (35.0)	163 (36.8)	**523 (32.2)**	<0.01

*P-value is based on Chi-square test for linear trend.

The discharge rate on antithrombotic therapy (STK-2) was 94.3% and 87.1% of eligible patients received thrombolytic therapy (STK-4) that met the standards set for performance measures. Similarly, for stroke education (STK-8) 88.8% of the patients met the standards set for performance measure. The Chi-square test for linear trend did not show any significant upward or downward trends in the performance measures over the 4 years. All data related to the performance measures are reported in Table 4.

**Table 4:** Summary statistics regarding performance measures of care for stroke patients in UTHSR by year, 2008-2011

**Performance measure**	**2008**			**2009**			**2010**			**2011**			**2008-2011**			***P*-value**
	**NE**	**NT**	**%**	**NE**	**NT**	**%**	**NE**	**NT**	**%**	**NE**	**NT**	**%**	**NE**	**NT**	**%**	
**STK-1**	744	713	95.8	803	783	97.5	859	845	98.4	949	934	98.4	3355	3275	97.6	0.98
**STK-2**	470	442	94.0	477	451	94.6	537	504	93.9	623	590	94.7	2107	1987	94.3	0.99
**STK-3**	77	62	80.5	63	61	96.8	77	75	97.4	65	64	98.5	282	262	92.9	0.81
STK-4*	70	63	90.0	68	56	82.4	93	81	87.1	88	78	88.6	319	278	87.1	0.99
**STK-5**	327	327	100	340	340	100	370	369	99.7	393	393	100	1430	1429	99.9	1.00
**STK-6**	326	271	83.1	331	285	86.1	340	300	88.2	409	355	86.8	1406	1211	86.1	0.96
STK-7**	680	663	97.5	659	639	97.0	710	639	90.0	819	707	86.3	2868	2648	92.3	0.29
**STK-8**	230	228	99.1	197	150	76.1	233	178	76.4	307	303	98.7	967	859	88.8	0.06
STK-9**	214	195	91.1	186	175	94.1	199	190	95.5	254	244	96.1	853	804	94.3	0.98
**STK-10**	635	607	95.6	684	657	96.1	735	709	96.5	880	860	97.7	2934	2833	96.6	0.99

*NE* = Number of eligible patients.

*NT* = Number of eligible patients who received treatment or intervention.

*P*-value = *P*-value based on Chi-square test for linear trend over four years, 2008–2011.

* Thrombolytic Therapy Administered (STK-4) is calculated based on inclusions and exclusions criteria set by The Joint Commission (TJC), which resulted in a different number of eligible patients for tPA compared to that reported in Table 2.

***The eligibility of patients related to diagnoses of ischemic stroke and hemorrhagic stroke:***[48,49]

We used the standard TJC definitions for inclusion/exclusion of patients for calculations of all performance measures. Patients with diagnosis of ischemic stroke are eligible for all performance measures, but patients with diagnosis of hemorrhagic stroke are eligible for performance measures STK-1, STK-7, STK-8, STK-9, and STK-10.

***Definition of performance measures***: [48,49]

*STK-1* = Venous Thromboembolism (VTE) Prophylaxis: Ischemic and hemorrhagic stroke patients who received VTE prophylaxis or have documentation why no VTE prophylaxis was given the day of or the day after hospital admission.

*STK-2* = Discharged on Antithrombotic Therapy: Ischemic stroke patients prescribed antithrombotic therapy at hospital discharge.

*STK-3* = Anticoagulation Therapy for Atrial Fibrillation/Flutter: Ischemic stroke patients with atrial fibrillation/flutter who are prescribed anticoagulation therapy at hospital discharge.

*STK-4* = Thrombolytic Therapy: Acute ischemic stroke patients who arrive at this hospital within 2 hours of time last known well and for whom IV t-PA was initiated at this hospital within 3 hours of time last known well.

*STK-5* = Antithrombotic Therapy By End of Hospital Day 2: Ischemic stroke patients administered antithrombotic therapy by the end of hospital day 2.

*STK-6* = Discharged on Statin Medication: Ischemic stroke patients with LDL greater than or equal to 100 mg/dL, or LDL not measured, or who were on a lipid-lowering medication prior to hospital arrival are prescribed statin medication at hospital discharge.

*STK-7* = Dysphagia Screening: Patients with ischemic or hemorrhagic stroke who undergo evidence-based bedside testing protocol approved by the hospital before being given any food fluids, or medication by mouth.

*STK-8* = Stroke Education: Ischemic or hemorrhagic stroke patients or their caregivers who were given educational materials during the hospital stay addressing all of the following: activation of emergency medical system, need for follow-up after discharge, medications prescribed at discharge, risk factors for stroke, and warning signs and symptoms of stroke.

*STK-9* = Smoking Cessation/Advice/Counseling: Patients with ischemic or hemorrhagic stroke with a history of smoking cigarettes, who are, or whose caregivers are, given smoking cessation advice or counseling during hospital stay. For the purposes of this measure, a smoker is defined as someone who has smoked cigarettes anytime during the year prior to hospital arrival.

*STK-10* = Assessed for Rehabilitation: Ischemic or hemorrhagic stroke patients who were assessed for rehabilitation services.

** Calculations for measures STK-7 and STK-9 are based on definitions provided in Disease-Specific Care Certification Program STROKE Performance Measurement Implementation Guide (2008) [49].

We observed an excellent IRR (Kappa ≥ 0.75) between the two abstractors for most categorical variables including diagnosis of stroke as infarct and ICH, tPA therapy, disposition, and initial presentation. IRR was moderate (0.40 ≤ Kappa < 0.75) for diagnosis of stroke as TIA. For most continuous variables, we also observed an excellent IRR (ICC ≥ 0.75), including age, onset time, arrival time, INR and mRS on discharge, except for IRR of tPA time that was poor (−0.48). Similarly, when we assessed the validity of categorical variables abstracted by each abstractor against the “gold standard”, for both abstractors we observed at least 93.3% agreement. For both abstractors, we also found 100 % sensitivity for these variables, except for initial presentation by abstractor #2 that had sensitivity of 95.4%. Specificity was 100% for all categorical variables except for disposition at “home” and “skilled nursing facility” for both abstractors that had specificity of 91.3% and 96.6%, respectively. For continuous variables, for both abstractors we observed a correlation of at least 0.83 for all variables, except for tPA time and INR for abstractor #2 that had correlation coefficients of −0.49 and 0.39, respectively. For continuous variables, we also reported mean differences. All data regarding the validity of the abstraction by the two abstractors against the “gold standard” are summarized in Table 5.

**Table 5:** IRR between the two abstractors, and validity against the “gold standard” for select variables, (N=30)

**Categorical variables**	**Dichotomous variables**	**IRR = Kappa (95% LCL)**^a^				**Abstractor 1 vs. “gold standard”**					**Abstractor 2 vs. “gold standard”**				
		**N**	**Abstractor 1 vs. Abstractor 2**	**Bias Index**		**N**	**Agreement (%)**	**Sensitivity (%)**	**Specificity (%)**	**Bias Index**	**N**	**Agreement (%)**	**Sensitivity (%)**	**Specificity (%)**	**Bias Index**
**Stroke type**	Infarct	30	1.00	0		30	100	100	100	0	30	100	100	100	0
	ICH	30	1.00	0		30	100	100	100	0	30	100	100	100	0
	TIA	30	0.65 (0.02)	−0.03		30	100	100	100	0	30	96.7	100	96.6	0.03
**tPA therapy**	Yes	30	1.00	0		30	100	100	100	0	30	100	100	100	0
**Symptomatic hemorrhage**	Yes	30	1.00	0		30	100	100	100	0	30	100	100	100	0
**Disposition**	Death	30	1.00	0		30	100	100	100	0	30	100	100	100	0
	Home	30	1.00	0		30	93.3	100	91.3	0.07	30	93.3	100	91.3	0.07
	Inpatient rehabilitation	30	1.00	0		30	100	100	100	0	30	100	100	100	0
	Skilled nursing facility	30	1.00	0		30	96.7	100	96.6	0.03	30	96.7	100	96.6	0.03
**Initial presentation**	MHH	30	0.92 (0.76)	0.03		30	100	100	100	0	30	96.7	95.4	100	−0.03
**Continuous variables**		**IRR = ICC**^d^**(95%LCL)**^a^				**Abstractor 1 vs. “gold standard”**					**Abstractor 2 vs. “gold standard”**				
		**N**	**Abstractor 1 vs. Abstractor 2**	**MD**^c^**(95%CI)**^b^		**N**	**Agreement (%)**	**Correlation (95%CI)**^b^	**MD**^c^**(95%CI)**^b^		**N**	**Agreement (%)**	**Correlation (95%CI)**^b^	**MD**^c^**(95%CI)**^b^	
**Age (years)**		30	1.00	0 (0, 0)		30	96.7	0.83 (0.65, 0.91)	1.4 (−1.5, 4.4)		30	96.7	0.83 (0.65, 0.91)	1.4 (−1.5, 4.4)	
**Onset time (minutes)**		16	1.00	4.7 (−14.5, 5.3)		16	93.8	1.00	9.4 (−10.6, -29.4)		16	87.5	1.00	14.1 (−7.7, 35.8)	
**Arrival time (minutes)**		29	1.00	0 (0, 0)		30	86.7	1.00	−0.2 (−1.9, 1.5)		29	89.7	1.00	−0.2 (−2.1, 1.6)	
**tPA time (minutes)**		7	−0.48 (−0.89)	NR		7	100	1.00	0 (0, 0)		7	57.1	−0.49 (−0.90, 0.45)	NR	
**INR**		25	0.98 (0.96)	0.02 (−0.02, 0.06)		25	80	0.98 (0.97, 0.99)	−0.01 (−0.04, 0.02)		26	73.1	0.39 (−0.00, 0.67)	0.1 (−0.2, 0.4)	
**mRS on discharge**		23	1.00	0 (0, 0)		23	95.6	0.98 (0.96, 0.99)	−0.04 (−0.13, 0.05)		26	96.2	0.98 (0.96, 0.99)	−0.04 (−0.12, 0.04)	

^a^ 95% *LCL* = 95% Lower Confidence Limit; ^b^ 95% *CI* = 95% Confidence Interval; ^c^*MD* = Mean difference for reliability study is calculated based on abstractor #1 minus abstractor #2; for validity study the difference is based on abstractor #1 or abstractor #2 minus the “gold standard”; ^d^*ICC* = Intra-class correlation; *NR* = Not reported because calculations were misleading due to an error in re-abstraction by abstractor #2 who reported 3 wrong dates (i.e., a wrong day, month or year) out of 7 dates for patients who received tPA therapy, which resulted in a misleading mean difference.

For all categorical variables in UTHSR we observed an accuracy rate (% agreement with the “gold standard”) of at least 96.5%. While sensitivity and specificity were 100% for most binary variables, wake-up stroke had the lowest sensitivity (91.7%) and IA therapy had the lowest specificity (92.9%). For continuous variables, we also observed a high level of agreement (>94.2%) for most variables, except for some date/time variables including CT time and arrival time that had accuracy rates of 80.8% and 85.2%, respectively. We also found very high correlations (r>0.96) between UTHSR records and the “gold standard” for almost all continuous variables, except for Glucose that had r=0.86. Mean differences for these variables between UTHSR records and the “gold standard” are reported in Table 6.

**Table 6:** Measures of validity of UTHSR data against “gold standard” based on 115 patient records, 2008–2011

**Categorical variables**	**Dichotomous variables**	**UTHSR vs. “gold standard”**				
		F*	**N**	**Agreement (%)**	**Sensitivity (%) (95% CI)**✪	**Specificity (%) (95% CI)**✪
**Gender**	Male	43	85	100	100 (92.0, 100)	100 (92.0, 100)
**Stroke type**	Infarct	62	115	100	100 (94.2, 100)	100 (93.3, 100)
	ICH	31	115	100	100 (88.8, 100)	100 (95.7, 100)
	TIA	2	115	100	100 (15.8, 100)	100 (96.8, 100)
**Hospital arrival mode**	Air	35	115	97.4	97.2 (85.5, 99.9)	97.5 (91.2, 99.7)
	Ambulance	59	115	97.4	95.2 (86.5, 99.0)	100 (93.3, 100)
**tPA therapy**	Yes	25	115	100	100 (86.3, 100)	100 (96.0, 100)
**tPA location**	MHH-TMC	13	25	100	100 (75.3, 100)	100 (73.5, 100)
**IA therapy**	No	13	115	98.3	99.0 (94.6, 99.9)	92.9 (66.1, 99.8)
**Atrial fibrillation in hospital**	Yes	19	115	99.1	99.0 (94.6, 99.9)	99.0 (94.3, 99.9)
**Anti-thrombotic medication**	Yes	52	78	100	100 (82.4, 100)	100 (86.8, 100)
**DVT prophylaxis**	Yes	78	85	100	100 (95.4, 100)	100 (59.0, 100)
**Patient education**	Yes	63	78	100	100 (94.3, 100)	100 (78.2, 100)
**Wake up stroke**	Yes	11	78	98.7	91.7 (61.5, 99.8)	100 (94.6, 100)
**Rehabilitation evaluation**	Yes	67	78	100	100 (94.6, 100)	100 (72.0, 100)
**Symptomatic hemorrhage**	Yes	2	115	100	100 (15.8, 100)	100 (96.8, 100)
**Disposition at discharge**	Death	17	115	100	100 (80.5, 100)	100 (96.3, 100)
	Home	43	115	98.3	100 (91.8, 100)	97.2 (90.3, 99.7)
	Inpatient rehabilitation	18	115	100	100 (81.5, 100)	100 (96.3, 100)
	Skilled nursing facility	4	111	96.5	NR	100 (99.9, 100)
**Initial presentation**	MHH	76	115	99.1	100 (95.3, 100)	97.4 (86.5, 99.9)
**Smoker**	Yes	20	78	100	100 (83.2, 100)	100 (94.0, 100)
**Continuous variables**			**N**	**Agreement (%)**	**Correlation (95% CI)**✪	**MD**✝ **(95% CI)**✪
**Age (years)**			115	98.3	0.96 (0.94, 0.97)	0.5 (−0.3, 1.2)
**Onset time (minutes)**			72	97.2	1.00 (1.00, 1.00)	6.3 (−3.0, 15.5)
**Arrival time (minutes)**			115	85.2	1.00 (1.00, 1.00)	26.7 (−36.3, 89.6)
**tPA time (minutes)**			23	95.7	1.00 (1.00, 1.00)	−0.04 (−0.13, 0.05)
**CT Time (minutes)**			52	80.8	1.00 (1.00, 1.00)	20.9 (−19.7, 61.4)
**Glucose**			78	96.2	0.86 (0.79, 0.91)	−5.9 (−15.6, 3.8)
**Creatinine**			78	98.7	0.99 (0.99, 1.00)	0.003 (−0.003, 0.008)
**LDL**			86	97.7	0.96 (0.94, 0.98)	114.9 (−113.5, 343.3)
**INR**			104	94.2	0.99 (0.99, 1.00)	−0.003 (−0.013, 0.006)
**mRS on discharge**			104	98.1	0.99 (0.99, 1.00)	0 (−0.03, 0.03)

***N*** = Where both UTHSR and gold standard data have values; ✪ 95% *CI* = 95% Confidence Interval; ✝ *MD* = Mean Difference, (calculated as UTHSR minus the “gold standard”).

*NR* = Not reported because there were no observations in UTHSR in the category of “Skilled Nursing Facility”; * *F* = Frequency of observations for each level of dichotomous variable reported.

**Figure 2:**
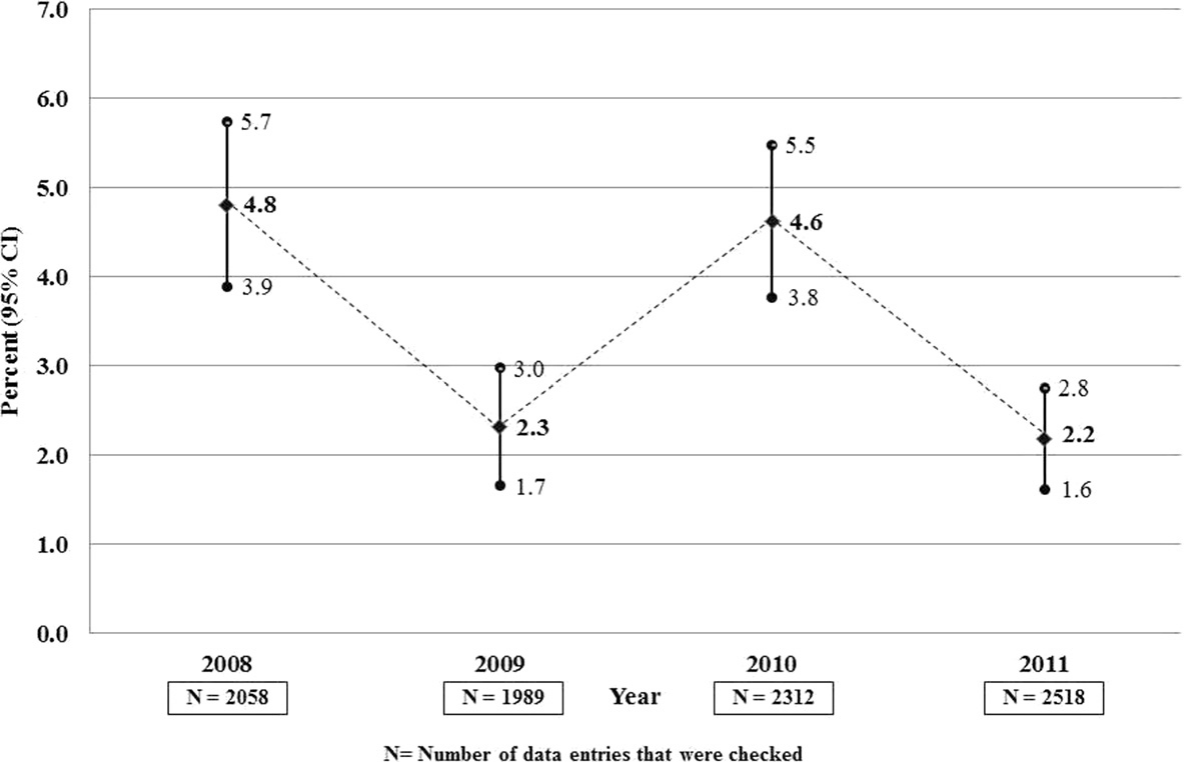
Estimated error rate (%) in UTHSR data with 95% CIs by year during 2008–2011.

Finally, our analysis indicated error rates of 4.8% [95% CI (3.9, 5.7)], 2.3% [95% CI (1.7, 3.0)], 4.6% [95% CI (3.8, 5.5)], and 2.2% [95% CI (1.6, 2.8)] for years 2008, 2009, 2010, and 2011, respectively. Furthermore, the differences between error rate in 2008 and subsequent years were statistically significant (all *P* < 0.001), except for the difference between 2008 and 2010. The numbers of data points used for calculation of error rates and 95% CIs for each of the four years, 2008–2011, are displayed in Figure 2.

## Discussion

In this article, we describe the development and assessment of enhanced quality assurance procedures in UTHSR and compare data quality in UTHSR before and after implementation of our enhanced QA procedures. Since we implemented our enhanced QA procedures for UTHSR at the end of 2008 when we received the second round of funding for SPOTRIAS, we believe 2008 serves as an important reference point and any potential improvements in our registry data would be reflected from 2009 and thereafter. Our finding of a significant reduction in UTHSR error rate from 4.8% in 2008 to 2.2% in 2011 (*P* < 0.0001) indicates that our effort in enhancing and formalizing the QA procedures has been successful in reducing the error rate. Though we observed an increase in error rate from 2.3% in 2009 to 4.6% in 2010 (*P* < 0.0001), we believe this is partly attributed to recruitment of two new chart abstractors in early 2010.

Other stroke registries have published data from different parts of the world [2,3,6,9,10,12,16-23,25], [27,28,54]. However, limited data have been reported regarding data quality as well as data quality assurance procedures for stroke registries. In fact, among the fourteen stroke registries that have published articles in English for which we had access to the full articles [2,3,5,6,10,13,14,16-19], [21,24,25,27-30,54,55], we found that only seven (50%) reported on the data quality of the data in their registries [2,5,10,13,14,16,18,24], [29,54,55]. The remaining seven registries did not discuss their data quality assurance procedures [3,17,19,21,25,27,28]. Since one of the aims of developing stroke registries is to improve the quality of care, and to reduce mortality attributable to stroke [6], it is imperative that the data used for such assessments is of high quality. Therefore, we devote a significant portion of the discussion in this paper to the enhanced QA procedures that we have established for ensuring data quality from chart abstraction to data management and analysis.

### Training of abstractors and assessment of reliability and validity of abstracted data

Reeves *et al.* (2008) has highlighted the importance of training in maintaining data quality for PCNASR registries [29]. Our quality assurance process rests on an understanding that each new abstractor should be provided sufficient training for codebook definitions and specialty issues of stroke (e.g., CT time). Historically, our abstractors used to be healthcare professionals including nurse practitioners; only recently, we have hired non-clinicians. This makes a difference in the level of training that needs to be done. To ensure reliability and validity of data, we believe training of abstractors should be mandatory [29].

The complexity and differences in the interpretation of the key variables in medical records for stroke patients demands continuous training and evaluation of the work conducted by the abstractors to ensure data quality in a stroke registry. For evaluation of data abstracted, we have conducted a reliability study to assess IRR between the two abstractors and a validity study to assess the level of agreement, sensitivity, and specificity between the data abstracted by each abstractor and the “gold standard”. For these evaluations, we have only used select variables from a set of 30 patient records for which the “gold standard” data were available.

We found excellent IRR (ICC ≥ 0.75) between the two abstractors for most continuous variables including age, onset time, arrival time, and mRS on discharge. These findings are consistent with those reported by Reeves *et al.* (2008) for PCNASR that indicated excellent reliability for age, stroke onset time, ED arrival time, and mRS [29]. Our findings are also consistent with those reported by Xian *et al.* (2012) for GWTG-Stroke registry indicating an excellent IRR for age [5]. We also found poor IRR (ICC = −0.48) for tPA time with a large mean difference between the two abstractors caused by discrepancies in dates (i.e., a wrong day, month, or year) for 3 patients out of 7 dates who received tPA therapy.

For categorical variables, we observed excellent IRR (Kappa ≥ 0.75) between the two abstractors for most variables including diagnosis of stroke as infarct and ICH, tPA therapy, disposition, and initial presentation, except for diagnosis of stroke as TIA that had a moderate IRR (Kappa = 0.65). These findings are consistent with those reported by Xian *et al.* (2012) for GWTG-Stroke registry that indicated excellent IRR for final clinical diagnosis, tPA therapy and discharge destination [5]. However, in PCNASR, Reeves *et al.* (2008) found poor reliability for stroke team consultation, time of initial brain imaging, discharge destination, and stroke/TIA diagnosed in emergency department [29]. Nonetheless, excellent IRR between the two abstractors alone does not indicate the validity of data abstracted. We believe assessment of both IRR and validity (% agreement, sensitivity, and specificity) provide a more informed evaluation of abstractors’ performance.

Our validity study based on 30 patient records revealed that for all selected categorical variables, the agreement between the data abstracted by each of the two abstractors against the “gold standard” was above 93.3%. Since the level of agreement alone does not indicate the validity of the data abstracted, we also computed bias index between each of the two abstractors and the “gold standard”. For example, for initial presentation at MHH-TMC we observed an agreement of 96.7% for abstractor #2 but the bias index for this variable against the “gold standard” was −0.03, indicating the tendency for abstractor #2 to record initial presentation as “other hospitals” while the “gold standard” indicated initial presentation at MHH-TMC. For continuous variables, we found a correlation of at least 0.83 for all selected variables, except for tPA time (r = −0.49) and INR (r = 0.39) for abstractor #2. Because a high correlation alone does not indicate the validity of the data abstracted, we also computed mean differences for these variables between each of the two abstractors and the “gold standard”. For example, for onset time we observed a perfect correlation (r =1) for both abstractors but the mean differences for this variable against the “gold standard” was 9.4 minutes for abstractor #1 and 14.1 minutes for abstractor #2. Interpretation of mean differences is similar to that of the bias index as described earlier. The 95% CIs for mean differences indicate no significant differences between the abstracted data by the two abstractors and the “gold standard”, except for tPA time for abstractor #2 that 95% CI is not reported. This is because abstractor #2 reported 3 wrong dates (i.e., a wrong day, month or year) out of 7 dates for patients who received tPA therapy, which resulted in a misleading mean difference. These findings indicate areas where additional training may be needed for the abstractors. Xian *et al*. (2012) used the audit abstractor for assessment of validity and accuracy of abstractors’ work against the audit data in GWTG-Stroke registry. They also reported a high accuracy rate for the majority of variables such as age, diagnosis and evaluation, arrival date/time, tPA therapy [5]. We believe an objective evaluation of the abstractors’ performance and providing additional training will result in improved data quality.

### Data cleaning

Data cleaning is an essential component of QA processes which includes identification and resolution of all discrepant data in the database [37,44]. However, the extent of data cleaning varies for different registries that reported their QA procedure [2,5,13,18,24]. As mentioned earlier, for UTHSR we developed a comprehensive program that included about 350 univariable and multivariable validation rules that were used to identify discrepant data. Whereas for GWTG-Stroke registry, the data abstraction tool included predefined logic features and user alerts to identify potentially invalid format or data entry. Required fields were structured so that valid data must be entered before the data are saved. Range checks were used for inconsistent or out-of-range data and prompted the user to correct or review data entries that were considered out of range [13]. Similarly, Hsieh *et al.* (2010) reported using logic checks and variable limits to prevent inaccurate data entries in TSR [2]. Since the data cleaning process does not capture all discrepant data, some recommend a random audit of a fraction of data in a stroke registry. For example, Hsieh *et al.* (2010) used random auditing of 5% of all cases entered in TSR [2].

#### Assessment of validity for data in UTHSR

It is important to note that accuracy of data is dependent on the type (e.g., categorical or continuous) and complexity of capturing accurate values for variables in a registry. For example, some registries have reported lower levels of accuracy for variables that involve date/time (e.g., time to an event) [56]. Therefore, it is important to assess validity of data for key variables in a stroke registry.

Our assessment of validity of data against the “gold standard” for categorical variables, based on 115 patient records for key variables and 85 patient records for the remaining variables, indicates an excellent agreement (> 96%) between the UTHSR data against the “gold standard”. Our findings are consistent with those reported by Xian *et al.* (2012), indicating high levels of agreement between audited data and medical records for categorical variables, except for DVT prophylaxis that had 79% agreement [5]. In addition, levels of sensitivity and specificity for categorical variables in UTHSR were excellent (above 92.9%). For continuous variables, we found a correlation of at least 0.86 for all selected variables. However, agreement for CT time and arrival time were 80.8% and 85.2%, respectively. For reasons described earlier, we also computed mean difference for these variables between the UTHSR and the “gold standard”. For example, for arrival time we observed a perfect correlation (r =1) for UTHSR but the mean difference for this variable against the “gold standard” was 26.7 [95% CI (−36.3, 89.6)] minutes. Therefore, specific attention needs to be made regarding CT time or arrival time because wrong dates (i.e., a wrong day, month or year) or sometimes error due to using different sources for capturing these variables could result in mean difference between the abstracted data by the abstractors and the “gold standard”. Others have also reported difficulty in capturing time related variables in stroke registries. For example, George *et al*. (2009) reported that for the majority (57.8%) of patients in PCNASR, the time from onset of symptoms to hospital arrival was not recorded or was not known [6]. Xian, *et al.* (2012) also reported on validity of data for continuous variables in GWTG-Stroke registry indicating accuracy rates of at least 85% for majority of variables, notably an agreement of more than 93.6 for arrival time and brain imaging time. However, their higher levels of agreement for the arrival and brain imaging times could be due to their definition of agreement for continuous variables, as they consider such data accurate if the values in the registry and in the audited records are within 15 minutes of each other [5], whereas we considered a perfect agreement for these variables in our study.

During 2008–2011, for UTHSR we observed a significant improvement in the error rate (dropped from 4.8% in 2008 to 2.2% in 2011). We attribute this improvement to our effort in enhancing and formalizing our quality assurance procedures that include enhancements in software development, improving instructions in the codebook, training of abstractors, evaluating reliability between abstractors, data cleaning, and assessment of validity of data in UTHSR. A few stroke registries have published accuracy rates or error rates as indicators of the overall validity of data in their registries [5,16]. For example, Asplund *et al*. (2011) reported an accuracy rate of at least 95% between the medical charts and data recorded in Riks-Stroke registry for stroke subtype and clinical data, but somewhat lower (approximately 85%) for data related to the healthcare organization at the participating hospitals [16]. Similarly, Xian *et al*. (2012) also reported 96.1% accuracy rate for data quality of GWTG-Stroke registry [5]. These published error rates are similar to that of our 2008 data from UTHSR, before our enhanced data quality assurance procedures were implemented. However, to our knowledge, our overall error rate in 2011 of 2.2% in UTHSR appears to be the lowest among all published error rates from stroke registries so far.

There are no established acceptable error rates for key variables in stroke registries, but for other epidemiologic and clinical trials the CDC recommends an error rate of 0.3% (3 per 1,000 entries) [57], which is usually achieved by conducting double data entry. Others have reported a more liberal estimate for the error rate, 1% to 5% for general databases used by many companies. However, 0.1% to 0.5% error rate is acceptable for clinical trials [58,59]. Studies that used single data entry procedures reported higher error rates compared with those who used double data entry [60]. Since the source of data error in stroke registries could be due to an error in abstraction or data entry, in order to achieve a significant reduction in the error rate, one should perform double data abstraction as well as double data entry. The decision whether to use a double data entry or single data entry process largely depends upon the availability of resources. For these reasons, some registries employ a single data abstraction and data entry procedure along with some sort of quality assurance procedure by re-abstracting and entering a small fraction (5%) for all the records to assess data quality in stroke registries [2].

Since the main focus of this paper is to assess the effect of enhanced QA procedures on data quality in UTHSR, we limited our discussion of the data obtained on our patients in UTHSR. While UTHSR data are mostly consistent with other stroke registries for hospitalized stroke patients, we observed differences in some characteristics. For example, the mean age of patients in UTHSR is 62.7 years (median = 63), significantly younger than that of the patients in ASTRAL (median = 72.5) [21] and PCNASR in Michigan (mean = 70.9 years) [29]. Four other participating states (Georgia, Illinois, Massachusetts, and North Carolina) that contributed data to PCNASR during 2005–2007, reported a median age of 72 years, but Georgia had younger patients (median = 67 years) [6]. In our registry, 51.4% had infarcts and 24.6% had hemorrhagic stroke. PCNASR reported that 14% of their patients were hemorrhagic and 56-58% were ischemic stroke [6,29]. A very large number of patients with ICH who were transferred to MHH-TMC rather than presenting directly to the ED, could explain a relatively higher percent of higher ICH patients in UTHSR. Compared with most other registries, UTHSR has a higher rate of air transport (22.5%). For example, Austrian Stroke Unit Registry reported that only 4.1% of patients were transported by helicopter from 32 stroke units between 2003 and 2009 [61]. This difference could be due to the fact that for over 30 years the UT Stroke team and MHH-TMC have been providing medivac helicopter service (Life Flight®) to bring stroke patients quickly to our stroke unit. In addition, depending upon distance and stroke severity, many other hospitals also choose to fly transferred patients to MHH-TMC. Furthermore, for more than five years, our stroke team has utilized telemedicine to “reach out” to smaller hospitals that do not have stroke expertise.

In UTHSR, the distribution of NIHSS baseline (NIHSS on admission) score comprised 40% less than or equal to 4; 31.5% within 5–14; and 28.5% more than 14. Our result for less severe stroke (NIHSS baseline ≤ 4) is consistent with the Switzerland registry (ASTRAL) (40.8%) [21]. For patients with ICH, the median NIHSS score was twice that of NIHSS score of patients with ischemic stroke. In UTHSR, the percentage of eligible patients who received tPA treatment within 3 hours had an upward trend, 21.3% in 2008 which increased to 27.3% in 2011. The Joint Commission Disease-Specific Care (DSC) suggested 10 items as performance measures for stroke registries [48,49]. We did not find a significant linear trend in the performance measures during 2008–2011.

Finally, we recognize that implementation of the proposed enhanced QA procedures may be cost prohibitive due to unavailability of the required resources for some institutions. We have been fortunate to have funding from SPOTRIAS that helped us to develop and implement these enhanced QA procedures in UTHSR. We believe that the utility of the proposed QA procedures is dependent on the objectives and use of data in stroke registries. Therefore, stroke centers that have or are planning to establish a stroke registry should carefully evaluate their needs and implement a suitable level of QA procedure to ensure reliability and validity of data.

##### Limitations

The decision to use 30 patient records for the reliability study (i.e., to assess IRR) was based on the following reports. First, Sim and Wright (2005) [62] have shown that sample size of 30 provides nearly 80% power for detecting an association between the two abstractors as long as Kappa ≥ 0.5. Second, Walter *et al.* (1998) have shown that sample size of greater than 22 provides at least 80% power for detecting any association between the two abstractors with respect to continuous measures as long as the ICC ≥ 0.5 [63]. However, we acknowledge a limitation that the sample of 30 for the “gold standard” records may not be sufficient to conduct inferential statistics on these data. Therefore, for assessment of validity of data in UTHSR, we have combined the 30 “gold standard” records with another 85 patient records for which information about all variables were re-abstracted and adjudicated by our data core team that included a vascular neurology clinician (faculty or fellow). This resulted in a total of 115 patient records used for assessing the validity of data in UTHSR. Since only select variables were included in the “gold standard” for the 30 patient records, the numbers of observations available for different variables in the validity study for UTHSR vary. Therefore, we recommend a careful interpretation of the data related to various inferences that could be made from these measures of validity. Overall, we believe the number of patient records used for the reliability and validity studies provide important information as to whether additional training is needed for any of the abstractors and for which variables such training is needed.

## Conclusions

In this study, we have described the UTHealth Stroke Registry and shown that establishment of enhanced data quality assurance has helped to improve the validity of our data. Our enhanced quality assurance procedures included training of abstractors, assessment of IRR between abstractors as well as assessment of validity of data abstracted compared with the “gold standard”, and development and implementation of univariable and multivariable data cleaning rules. We have observed an excellent inter-rater reliability and validity for almost all key variables. Our resulting data compare well with data from other registries and certification guidelines, and demonstrate tPA treatment rates that are among the highest reported.

## Competing interests

The authors declare that they have no competing interests.

## Authors’ contributions

MHR, NRG, MRS, and JCG have made substantial contributions to conception and study design; NRG, JCG, SIS, MRS, FSV, JDT, AT, AAD, RMM, and EEC contributed to acquisition of data; MHR, NRG, MRS, RP, AT, EEC, FSV, JDT, AAD, RMM have made contributions to data quality assurance procedures; MHR, HP, MAH, RP, and MRS conducted data analysis; MHR, NRG, JCG, and MAH have contributed to interpretation of data; MHR, MAH, and AT significantly contributed to drafting of the manuscript and NRG, JCG, SIS, MRS provided critical revision of the manuscript; All authors have read and approved the final version submitted for publication.

## Pre-publication history

The pre-publication history for this paper can be accessed here:

http://www.biomedcentral.com/1471-2377/13/61/prepub
